# Evaluating approved and alternative treatments against an oxytetracycline-resistant bacterium responsible for European foulbrood disease in honey bees

**DOI:** 10.1038/s41598-022-09796-4

**Published:** 2022-04-07

**Authors:** Fatima Masood, Jenna M. Thebeau, Allyssa Cloet, Ivanna V. Kozii, Michael W. Zabrodski, Sarah Biganski, Jenny Liang, M. Marta Guarna, Elemir Simko, Antonio Ruzzini, Sarah C. Wood

**Affiliations:** 1 Department of Veterinary Microbiology, University of Saskatchewan, Saskatoon, SK Canada; 2 Veterinary Pathology, University of Saskatchewan, Saskatoon, SK Canada; 3 Biochemistry, Microbiology and Immunology, University of Saskatchewan, Saskatoon, SK Canada; 4 Agriculture and Agri-Food Canada, Beaverlodge Research Farm, Beaverlodge, AB Canada; 5 Prairie Diagnostic Services Inc., Saskatoon, SK Canada

**Keywords:** Microbiology, Antimicrobials, Applied microbiology, Bacteria, Pathogens, Infection

## Abstract

European foulbrood (EFB) is a disease of honey bee larvae caused by *Melissococcus plutonius*. In North America, oxytetracycline (OTC) is approved to combat EFB disease though tylosin (TYL) and lincomycin (LMC) are also registered for use against American foulbrood disease. Herein, we report and characterize an OTC-resistant *M. plutonius* isolate from British Columbia, Canada, providing an antimicrobial sensitivity to the three approved antibiotics and studying their abilities to alter larval survival in an in vitro infection model. Specifically, we investigated OTC, TYL, and LMC as potential treatment options for EFB disease using laboratory-reared larvae infected with *M. plutonius*. The utility of the three antibiotics were compared through an experimental design that either mimicked metaphylaxis or antimicrobial intervention. At varying concentrations, all three antibiotics prevented clinical signs of EFB disease following infection with *M. plutonius* 2019BC1 in vitro. This included treatment with 100 μg/mL of OTC, a concentration that was ~ 3× the minimum inhibitory concentration measured to inhibit the strain in nutrient broth. Additionally, we noted high larval mortality in groups treated with doses of OTC corresponding to ~ 30× the dose required to eliminate bacterial growth in vitro. In contrast, TYL and LMC were not toxic to larvae at concentrations that exceed field use. As we continue to investigate antimicrobial resistance (AMR) profiles of *M. plutonius* from known EFB outbreaks, we expect a range of AMR phenotypes, reiterating the importance of expanding current therapeutic options along with alternative management practices to suppress this disease.

## Introduction

Honey bees (*Apis mellifera)* are the most economically significant pollinators of major crops worldwide^1^. It is estimated that roughly one third of food that is consumed is either directly or indirectly dependent on honey bee pollination^2^. Safeguarding honey bee health through the management of infectious diseases of honey bees is therefore critical to the success of global agriculture. In North America, antimicrobials are widely used for treatment and control of bacterial brood diseases, including both American foulbrood (AFB) and European foulbrood (EFB) disease. In contrast, prophylactic antimicrobial-use is prohibited in beekeeping in the European Union^3^. Thus, antimicrobial resistance (AMR) poses an imminent threat to North American apiaries, especially for cases of EFB disease since only a single antibiotic, oxytetracycline (OTC), is approved for use^4^. EFB is the result of an infection of honey bee larvae by the Gram-positive bacterium *Melissococcus plutonius,* occurring through the consumption of contaminated food provided by adult bees^5^. Adult bees are asymptomatic carriers that can transmit the pathogen within and between colonies. The high density of bee populations within colonies and apiaries, as well as the density of apiaries within a region, facilitates the transmission of *M. plutonius* to larvae, which can result in immense brood loss leading to weakened colonies and even colony collapse^5,6^. Upon ingestion, *M. plutonius* multiplies in the developing midgut and competition for nutrients between host and bacterium has been hypothesized to result in clinical symptoms of EFB disease that ultimately results in death through starvation^5,7^.

EFB disease has been reported worldwide and has recently seen a re-emergence in western Canada^8^. In this region, honey bee colonies have historically been characterized by a capacity to spontaneously recover from EFB disease, however, recent disease emergence has included unconventional cases and poor recovery without intervention^9^. The increase in honey bee colonies unable to spontaneously recover from EFB has translated not only to a disease state that is increasingly difficult to eradicate but also increased transmission^8^. These distinctly new cases of EFB disease are hypothesized to be caused by hypervirulent strains of *M. plutonius* circulating in western Canada. An alternative hypothesis posits that resistance to conventional antimicrobial therapies explains the recalcitrance of recent EFB disease outbreaks in western Canada. While some geno- and phenotypic characterization of *M. plutonius* has been reported from Europe and Asia, the bacteria responsible for recent outbreaks in Canada remain to be characterized at the same level^10,11^.

Currently, oxytetracycline (OTC) is the only approved antimicrobial used for treatment of EFB disease in Canada and it is applied to individual hives at a concentration of 200 mg per 20 g of icing sugar, for three consecutive doses, 4–5 days apart^4^. OTC is a bacterial natural product that was among the first discovered tetracyclines, a drug class that has been widely used in both human and veterinary medicine since the late 1940s^12^. The tetracyclines are broad-spectrum, bacteriostatic antibiotics that inhibit protein synthesis by preventing aminoacyl-tRNA attachment to the A site of the ribosome. Accordingly, resistance to OTC and other tetracyclines are now common in animal pathogens with a widespread distribution of AMR genes transferred through bacterial mechanisms of genetic exchange. These genes, which have a colloquial naming system based on discovery, can encode for a series of protective functions such as efflux, ribosomal modification proteins, and antibiotic destruction. In the context of honey bee health, OTC-resistance has been observed in *Paenibacillus larvae*, the causative agent of AFB disease, resulting from plasmid-encoded tetracycline resistance through the *tetL* gene^13^. Additionally, a series of *Bacillus cereus* strains isolated from honey were found to carry tetracycline resistance genes, including *tetL* and *tetK*^14^. These results indicate the presence of AMR genes in pathogens and non-pathogenic organisms within colonies that may act as reservoirs of tetracycline resistance genes.

The risk of *M. plutonius* developing resistance to OTC is heightened by its use to treat AFB disease^3^. Alternative treatments to suppress AFB disease are currently approved in Canada, including tylosin (TYL) and lincomycin (LMC). Notably, TYL is a macrolide antibiotic that is widely used in western Canada to treat livestock animals that are commonly raised near apiaries in the region^15^. LMC is a lincosamide antibiotic that, like the macrolides, inhibit protein synthesis by binding to the 23S subunit of the ribosome^15–17^. Due to similarities in their mechanism, including overlapping drug binding sites, bacteria can develop cross-resistance to both TYL and LMC via macrolides, lincosamides, and streptogramin B (MLS_B_) resistance^18,19^. MLS_B_ resistance is typically associated with genes that encode for methyltransferases that modify a common target site of the antibiotic. For example, *erm* genes, a major source of MLS_B_ resistance, encode for rRNA methyltransferases that add one or two methyl groups onto the 23S rRNA, preventing the antibiotics from binding their target^18^. While no TYL or LMC-resistant *P. larvae* or *M. plutonius* have been identified, bacteria isolated from honey encode for MLS_B_ resistance genes, including plasmid-borne 23S rRNA methyltransferases such as *ermC*^13^*.* Additionally, a recent study found *M. plutonius* isolates resistant to the macrolide antibiotic, mirosamicin^20^. Thus, there exists a possibility of *M. plutonius* developing MLS_B_ resistance. Antimicrobial use in honey bees has been shown to negatively impact the honey bee microbiome, leading to dysbiosis^21–23^. Microbial imbalance can lead to changes in gene expression, metabolism, and allow for the overgrowth of opportunistic pathogens. Nevertheless, antibiotic use can be beneficial in the case of treating and preventing foulbrood diseases^23^.

In this study, we evaluated the potential utility of both EFB and AFB disease-approved treatments in protection against infection by *M. plutonius* using an in vitro model. Prophylactic treatment of EFB in honey bees in north America is prohibited^4^. Thus, our experimental design considered both metaphylaxis (concurrent infection and treatment) and therapeutic (treatment after infection) antimicrobial interventions of larvae infected with a previously described isolate from British Columbia named *M. plutonius* 2019BC1^24^. The minimum inhibitory concentration (MIC) and minimum bactericidal concentration (MBC) of the three antibiotics against *M. plutonius* 2019BC1 were determined and antibiotic concentrations within and above the range of environmental exposures were evaluated for their ability to protect against EFB and harm honey bee larvae in vitro. Our results are discussed within the context of EFB disease and the North American beekeeping industry.

## Materials and methods

### Determination of minimum inhibitory concentrations

The minimum inhibitory concentrations (MICs) of OTC (TCI, 00475), TYL (Thermo Scientific, AC463070050), and LMC (Thermo Scientific, AAJ6125106) were determined using both a broth microdilution method in a 96-well format and growth on an agar surface supplemented with antibiotics. KSBHI (brain heart infusion supplemented with 0.15 M KH_2_PO_4_ and 1% soluble starch)^25^ or KSBHI with 1.5% agar were used to support bacterial growth. For the 96-well formatted broth assay, antibiotics were prepared as twofold serial dilutions in 200 μL of KSBHI. Dilution series includes 128–2 μg/mL OTC and 16–0.25 μg/mL TYL or LMC. To measure the MICs, a culture of *M. plutonius* 2019BC1 was inoculated into KSBHI with and without antibiotic at an optical density of 5 × 10^−4^, measured at a wavelength of 600 nm. Growth was evaluated after a 72-h incubation in microaerophilic conditions (AnaeroPack System, Mitsubishi Gas Chemical Company, Inc.) at 37 °C, shaking at 200 rpm. The MIC was defined as the lowest concentration of antibiotic in which no *M. plutonius* growth was visible. For each MIC determination, three technical replicates were performed on three biological replicates of *M. plutonius* 2019BC1. For the agar plate method, antibiotics were also prepared as twofold serial dilutions in KSBHI agar as above, though 1 mL volumes were used to generate agar surfaces in a 48-well plate. A culture of *M. plutonius* 2019BC1 was diluted to an optical density of 5 × 10^−4^ before inoculating agar surfaces with 2 μL. Growth was evaluated after a 96-h incubation in microaerophilic conditions at 37 °C. For each MIC determination, three technical replicates were performed on three biological replicates of *M. plutonius* 2019BC1.

### Determination of minimum bactericidal concentrations

The minimum bactericidal concentrations (MBCs) of OTC, TYL, and LMC were determined by inoculating 2 mL of fresh, antibiotic-free KSBHI media with samples from 96-well MIC plates that had no visible *M. plutonius* growth after 72 h. The growth of surviving bacteria was then evaluated after a 72-h incubation in microaerophilic conditions at 37 °C, shaking at 200 rpm. The MBC was defined as the lowest concentration of antibiotic in which no *M. plutonius* growth was observed after 72 h in the fresh outgrowth media.

### Source of honey bee larvae

Six well-established, healthy, experimental field colonies in Saskatoon, SK, Canada, were used as the source of honey bee larvae. Freshly hatched larvae used for in vitro larval rearing experiments were generated by placing empty brood frames in a cage with a laying queen. Twenty-four hours later, frames were removed from the queen cages and placed in adjacent uncaged space in the brood chamber. After three days, the frames, which contained first-instar larvae, were transported to the laboratory in a portable incubator kept at 35 °C. Genetically unrelated queens were used as host genetics contribute to the outcome of the disease progression^26^.

### Larval rearing, infection and antimicrobial treatments in vitro

The in vitro larval rearing protocol was adapted from Schmehl et al.^27^. Field-collected larvae were transferred (grafted) from brood frames into 48-well sterile tissue culture plates (considered day 0, d0, of the experiment). Briefly, each well contained a 1 cm diameter cups and 10 μL of pre-warmed diet A (44.25% royal jelly, 5.3% each of glucose and fructose, 0.9% yeast extract, and 44.25% water), and were maintained at 35 °C throughout the grafting process. After grafting, an additional 9.5 μL of diet A mixed with either 0.5 μL PBS (used for the grafting control) or 0.5 μL PBS containing 50 CFU of *M. plutonius* 2019BC1 (sequence type 19, clonal complex 12), isolated from a honey bee colony with clinical signs of EFB in B.C, Canada, was added^24^. Plates were incubated at 35 °C in an environment humidified by 0.9 M K_2_SO_4_. Larvae were fed increasing amounts of diet throughout the experiment with the exception of d1 when no additional food was provided. Plates were monitored daily using a stereomicroscope, and dead larvae were removed immediately. Larval death was determined based on melanisation, the lack of mobility and spiracle movement^24^. Rearing experiments were terminated 6 days after the grafting date (d6).

Treatment and control groups were defined by dividing 48-well plates into four groups of 12 larvae. Each plate included a negative control group (grafting control; GC, n = 12 animals) consisting of uninfected larvae fed control diet. A minimum of two technical replicates (n = 24) and two to five biological replicates (experiments performed using different queens corresponding to distinct genetic lineages) were performed for each treatment group. Treatment groups included 1, 10, 100, and 1000 μg/mL of OTC, and 33, 330, and 3300 μg/mL of both TYL and LMC. To ensure that effects were not caused by variations in grafting (a common technical challenge) or potential poor larval health, only experimental plates with > 75% survival in the grafting controls were used in the study. Likewise, experimental infection was determined by using plates only when < 50% survival of the I0 control was observed.

Antibiotics were included in diets daily for either 4 or 5 days depending on the experimental design, Thus, total 4 or 5-day doses were of 0.14–140 μg or 0.16–160 μg OTC and 4.62–462 μg or 5.28–528 μg of TYL and LMC not accounting for the half-lives of OTC, TYL, and LMC, which in water are 34 h, 200 d, and 30 h, respectively^28–30^.

Statistical analyses were performed using Stata 17 with larval survival at d6 compared among treatment groups using a Pearson’s chi-square test.

### Measure of average healthy larval weight

On d6 of in vitro larval rearing trials, surviving larvae were rinsed with water to remove any excess diet before being gently patted dry. Dry larvae were then weighed using an analytical balance.

### Survival of *M. plutonius* in larval diets

*M. plutonius* 2019BC1 was diluted to 5000 CFU/mL in diet A, with or without antibiotics, to mimic preparations used for larval infection. The diets were incubated for 30 min at 35 °C and subsequently, 50 μL of each diet was plated, in triplicate, and grown in microaerophilic conditions at 37 °C for 72 h. The number of viable of *M. plutonius* were counted and reported as CFU/mL.

### Screening publicly available *M. plutonius* genomes for tet resistance genes

18 Publicly available *M. plutonius* genomes^10^, were downloaded from NCBI and uploaded to the Comprehensive Antibiotic Resistance Database (CARD) to identify known tetracycline resistance genes^31^.

## Results and discussion

### *M. plutonius* 2019BC1 is resistant to oxytetracycline

To define the *M. plutonius* 2019BC1 AMR phenotype in vitro, the three antibiotics of interest—OTC, TYL and LMC—were evaluated using two-fold serial dilutions in a microbroth dilution assay followed by antibiotic-free outgrowth to define the minimum inhibitory concentration (MIC) and minimum bactericidal concentration (MBC; Table 1). *M. plutonius* 2019BC1 was most sensitive to LMC (MIC 0.5 μg/mL), followed by TYL (2 μg/mL). Remarkably, the 2019BC1 isolate was resistant to OTC with an MIC of 32 μg/mL required to suppress visible growth of the strain in KSBHI broth. While *M. plutonius* is considered sensitive to OTC at a cut-off value of 2.5 μg/mL according to Clinical & Laboratory Standards (CLSI) guidelines, there is no information for what constitutes an OTC-resistant isolate^32^. Unlike *M. plutonius*, OTC-resistance in *Paenibacillus larvae*, which causes AFB disease, has been identified^33–35^. According to CLSI guidelines, *P. larvae* is considered resistant to OTC at ≥ 16 μg/mL using a broth microdilution method^32^. Thus, applying the threshold used to classify *P. larvae*, another honey bee pathogen, *M. plutonius* 2019BC1 can be classified as resistant to OTC. Alternatively, *M. plutonius* may be tolerant to high concentrations of OTC. Bacterial tolerance is defined as survival without replication in the presence of an antibiotic^36^. However, the MIC of a tolerant strain is typically similar to that of a susceptible strain, while the minimum time of killing would be longer for a tolerant strain than a susceptible strain^37^. Thus, the over 12-fold increase in MIC of *M. plutonius* 2019BC1 relative to the CLSI OTC susceptibility cut-off value of 2.5 μg/mL for *M. plutonius* suggests that *M. plutonius* 2019BC1 is an OTC-resistant strain^32^. To the best of our knowledge, this is the first report of an OTC-resistant strain of *M. plutonius* recovered from an EFB disease outbreak. Previous studies of *M. plutonius* isolates from the United Kingdom, where antimicrobial use in apiculture is prohibited, and Australia were sensitive to OTC at ~ 4 and 1–2 μg/mL, respectively^11,38^. These studies measured MICs on solid agar, prompting our own evaluation of the antibiotics in KSBHI. *M. plutonius* 2019BC1 was resistant to OTC at 16 μg/mL when grown on a solid KSBHI agar surface. The two-fold difference in MIC may have resulted from a difference in bacterial physiology during vegetative versus growth on a solid surface, though variations within a twofold dilution are typical in these types of experiments. In either case, extrapolating the CLSI guidelines for *P. larvae* to *M. plutonius*, the 2019BC1 isolate is resistant to OTC^32^.

**Table 1:** Susceptibility of *M. plutonius* 2019BC1 to antibiotics (μg/mL).

Antibiotic	MIC (agar)	MIC (broth)	MBC
OTC	16	32	128
TYL	1	2	4
LMC	0.5	0.5	2

Classification of *M. plutonius* 2019BC1 as OTC resistant is of particular interest. The resistant phenotype is an expected result considering the frequent use of OTC as a treatment for EFB and AFB in North America^4^. In fact, the concentration of OTC required to inhibit this strain in vitro is higher than the expected accumulation of the antibiotic during a standard treatment. For example, honey bee larvae sampled from one or two chamber field colonies, one day after colony treatment with 300 mg OTC (in excess of current label recommendations of 200 mg), were shown to have OTC concentrations of ~ 13 and 9 μg/g, respectively^39^. While this appears to be a sub-therapeutic dose for infection with *M. plutonius* 2019BC1, social immunity (see below) may influence on colony-level antibiotic efficacy. Nevertheless, this first observation of OTC-resistant *M. plutonius* calls for additional sampling and characterization of *M. plutonius* isolates from North American apiaries, including intensive AMR phenotyping and whole genome sequencing, to uncover the genetic determinants of antibiotic resistance in this pathogen. A survey of 18 other, previously assembled, publicly available *M. plutonius* genomes did not yield significant hits to any known tetracycline resistance genes, suggesting that further studies will need to be done to uncover the genetic determinants of OTC resistance in *M. plutonius.*

### Antibiotics prevent and control *M. plutonius* infections

**Figure 1:**
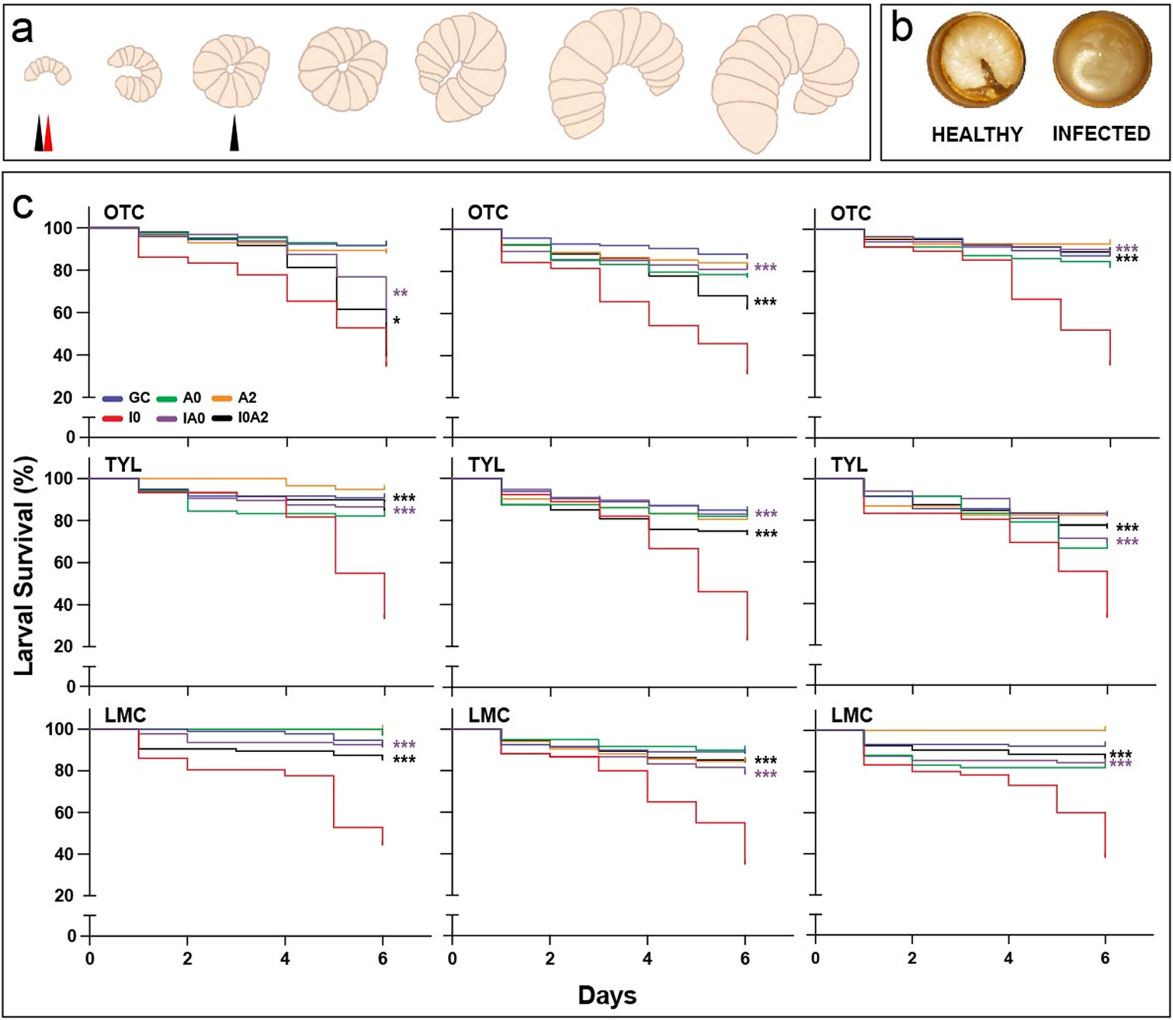
Results of in vitro larval rearing experiments upon exposure to *M. plutonius* with and without antibiotics. (**a**) Cartoon representation of typical growth of healthy larvae during a week-long in vitro rearing experiment. The red arrow indicates the day at which larval infection with 50 CFU of *M. plutonius* 2019BC1 occurred (d0), while the black arrows indicate the days on which an antibiotic was first administered (d0 or d2). (**b**) Representative phenotypes of healthy and infected larvae. Healthy d6 larvae are white and plump, while infected d6 larvae are typically smaller in size and typically darker in colour. (**c**) Survival of in vitro reared honey bee larvae based on Table 2, excluding 1000 μg/mL OTC. The top three panels, from left to right, display survival in experiments conducted with 1, 10, and 100 μg/mL OTC. The middle and bottom panels display survival in experiments with 33, 330, and 3300 μg/mL of TYL and LMC, respectively. Statistical analysis of the survival was performed using a chi-square two-way association test comparing either IA0 or I0A2 with the I0 and significant differences are denoted by asterisks (*p < 0.1, **p < 0.001, ***p < 0.0001).

To evaluate the effects of antibiotics on larval survival and bacterial infection, we designed an in vitro rearing experiment that included two distinct dosing strategies. The first strategy mimics metaphylactic treatment by administering antibiotics at the time of grafting (A0) or co-administration of antibiotic and *M. plutonius* at the time of grafting (IA0). The second strategy involved antimicrobial intervention two days after grafting (A2) or infection (I0A2). Rearing treatments were performed in parallel to demonstrate effects of grafting (GC) and untreated infection (I0). Larval infections were established using 50 CFU of *M. plutonius* 2019BC1 as previously described^24^. Growth of the larvae was monitored for 1 week: typical healthy and infected phenotypes show significant macroscopic differences during this timeframe (Fig. 1a, b). The average weight of healthy larva that survived the rearing experiment was 130 ± 20 mg (n = 517), in line with previous studies^26,40^. Experiments were conducted with a range of distinct genetic lineages with larva sourced from different colonies (Table 2). The concentrations of OTC were selected based on a previous work that tested its effects on honey bee larvae infected with *M. plutonius in vitro*^41^. The TYL concentrations were selected based on a previous study of the antibiotic treatment for AFB in vitro*,* which also considered field relevant concentrations^42^. The doses of LMC mirrored those of TYL due to the similar applications of these drugs for the treatment of AFB in the field^4^.

**Table 2:** Summary o**f** larval rearing experiments showing survival^a^ 6 days after grafting and treatment.

	No. of queens	No. of larvae	GC	I0	A0	IA0	A2	I0A2
			Grafting control	Infection control	Metaphylaxis control	Metaphylaxis treatment	Therapeutic control	Therapeutic treatment
1 µg/mL OTC	3	527	91 ± 5	28 ± 11	92 ± 10	56 ± 21	83 ± 13	40 ± 12
10 µg/mL OTC	2	571	86 ± 7	31 ± 15	77 ± 8	81 ± 9	82 ± 10	62 ± 13
100 µg/mL OTC	5	480	87 ± 12	35 ± 8	82 ± 11	86 ± 21	93 ± 10	88 ± 11
1000 µg/mL OTC	5	625	88 ± 9	33 ± 33	0 ± 0	0 ± 0	9 ± 12	8 ± 9
33 µg/mL TYL	5	431	90 ± 6	25 ± 7	82 ± 16	83 ± 13	95 ± 5	85 ± 15
330 µg/mL TYL	4	667	84 ± 10	23 ± 14	82 ± 18	82 ± 14	81 ± 10	73 ± 13
3300 µg/mL TYL	3	324	82 ± 7	33 ± 8	67 ± 35	69 ± 15	82 ± 11	76 ± 13
33 µg/mL LMC	2	383	92 ± 8	44 ± 5	97 ± 5	92 ± 6	100 ± 0	85 ± 7
330 µg/mL LMC	4	481	88 ± 9	35 ± 11	90 ± 4	78 ± 7	85 ± 11	84 ± 5
3300 µg/mL LMC	3	381	91 ± 7	22 ± 5	81 ± 11	86 ± 10	100 ± 0	86 ± 21

^a^Survival is expressed as mean percent ± standard deviation between experiments.

All three antibiotics were able to reduce larval mortality from *M. plutonius* infection at their low, medium, and high concentrations (Fig. 1c, Table 2). Antibiotic treatment significantly increased larval survival from *M. plutonius* infection by 12–53%, 36–60%, and 41–64% relative to the infection control group (I0) for OTC, TYL, and LMC, respectively (Fig. 1c; *p < 0.1, **p < 0.001, and ***p < 0.0001). We did not observe lineage-specific responses in our assays. This is the first in vitro demonstration of the efficacy of TYL or LMC as treatments against *M. plutonius* infection in honey bee larvae whereas OTC has been previously shown to be effective in treating *M. plutonius* infection of in vitro-reared larvae^41^.

In this previous study, OTC concentrations ranging from 1 to 10 μg/mL reduced larval mortality from *M. plutonius* infection, and treatment with 20 μg/mL OTC was required to reduce larval mortality to the same level as the negative control (non-infected larvae); however, the AMR phenotype of this strain (e.g. MIC of OTC) used was not evaluated^41^. Here we show that concentrations of 1 μg/mL and 10 μg/mL OTC were able to significantly increase larval survival by 12–50% when compared to an infected control (I0; *p < 0.1, **p < 0.001, ***p < 0.0001; Fig. 1c), though survival of these groups were significantly lower by 5–51% relative to the uninfected GC group (*p* < 0.0001; Table 2). Larval treatment with 100 μg/mL OTC, corresponding to ~ 3× the MIC in vitro, was necessary to reduce larval mortality to the same level as the uninfected GC group. After six days of in vitro rearing, larvae treated with 100 μg/mL OTC were calculated to receive a total dose of 105–120 μg/g OTC per larvae, not accounting for the 34-h half-life of OTC in water^28^. Accordingly, the dose of OTC per larva in vitro is ~ 10× the quantity of OTC previously reported in honey bee larvae from a field colony treated with 300 mg OTC^39^. In the field, we anticipate that concentrations lower than 100 μg/mL will be sufficient due to the presence of social immunity within a colony as a mechanism for pathogen defense. Examples of honey bee social immunity involves the production of antimicrobial peptides, such as defensin-1, grooming, and hygienic behaviour, which refers to the colony’s ability to detect and remove diseased brood^43^.

In contrast to the protection provided by the 100 μg/mL dose of OTC, we observed significant toxicity to larval brood from the antibiotic at 1 mg/mL. This high dose resulted in nearly complete killing of the larvae with only 0–8% larval survival observed in the antimicrobial treatment groups. This observation is well-aligned with anecdotal evidence communicated by beekeepers reporting OTC-dependent brood toxicity within colonies. Similarly, previous controlled experiments have demonstrated that OTC can be toxic to honey bee larvae when administered to a colony in a 5 mg/mL sucrose solution or as a powder treatment according to label instructions at 200 mg OTC per 20 g of icing sugar^44,45^.

### Larval diet inhibits *M. plutonius* survival

**Figure 2:**
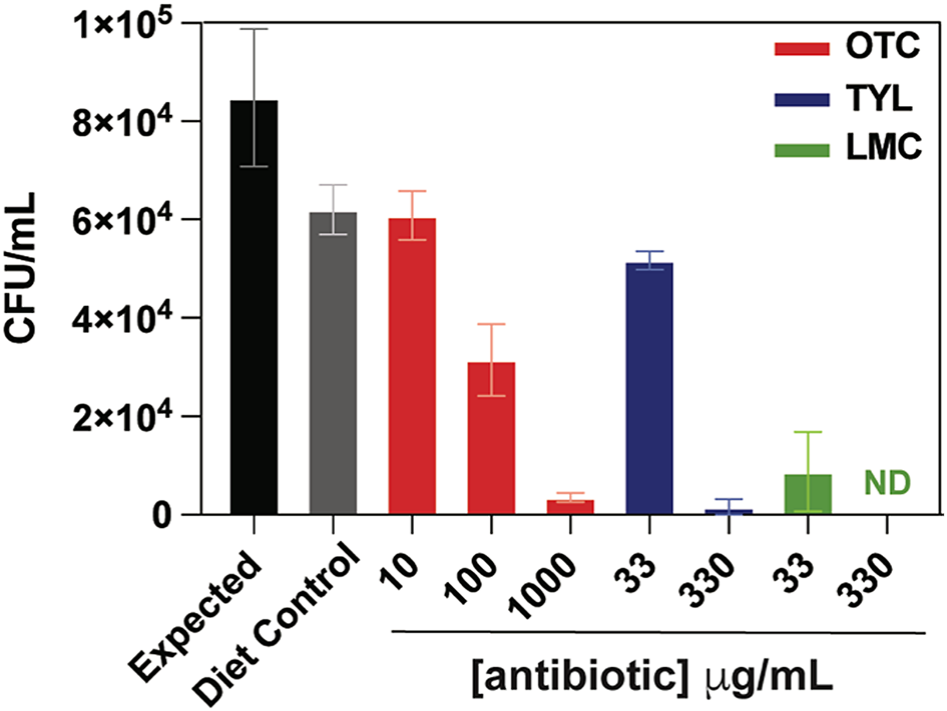
Survival of *M. plutonius* in larval diet and diets supplemented with antibiotics. Recovery of *M. plutonius* 2019BC1 from diets is presented in CFU/mL after a 30 min incubation in control larval diet A (grey) or larval diet A containing various concentrations of OTC (red), TYL (blue), or LMC (green). Expected (black) represents expected recovery of *M. plutonius* in KSBHI with no antibiotic or diet component. The mean and standard deviations are plotted. The detection limit was 20 CFU/mL. ND indicated no detection.

While the larval rearing experiment provided information related to metaphylactic and curative effects of antibiotics delivered to larvae, we sought to better understand the ability of each drug to affect *M. plutonius* in the laboratory diet. Moreover, our laboratory infection model is atypical in that relatively few CFUs are delivered to larvae in order to establish disease when compared to others^41,46^. Thus, to clearly establish the consequences of concurrent administration of antimicrobials and *M. plutonius* during our in vitro rearing experiments, we inoculated antibiotic-containing and antibiotic-free diets with *M. plutonius* for 30 min at 35 °C before recovering viable bacteria on KSBHI agar (Fig. 2). In all cases, *M. plutonius* viability decreased, including a 27% loss of *M. plutonius* counts within the antibiotic-free, control diet relative to the expected values of *M. plutonius* in KSBHI. The larval diet itself possesses modest antimicrobial activity and has been previously shown to inhibit *M. plutonius* growth^47,48^. Recently, the antimicrobial activity of the diet has been linked to Royal Jelly, and more specifically, Major Royal Jelly protein 1 (MRJP1) was shown to be the determinant of *M. plutonius* inhibition^49^. The addition of low doses of TYL and OTC to diets showed similar reductions in *M. plutonius* viability compared to the control diet, while greater 63–98% reductions in *M. plutonius* were observed in diets containing concentrations of 100 μg/mL OTC and 330 μg/mL TYL. LMC treatment was associated with the most dramatic declines in *M. plutonius* survival in the diet, mirroring the observed MICs in vitro. At 330 μg/mL LMC, no viable *M. plutonius* were recovered from the diet after 30 min, representing at least a 4-log reduction in the bacterium, considering the detection limit of this assay is 20 CFU/mL (Fig. 2). Due to the reduction of *M. plutonius* viability in larval diet, co-administration of high doses of antibiotics and bacteria on d0 of in vitro larval rearing (Fig. 1a) likely resulted in a lack of larval infection*.* Indeed, the effect of the diet, and its preparation with and without antibiotics, may explain the need in other models of EFB to establish larval infection in vitro with doses as high as 10^7^ CFU^41,46^. However, CC12 infections require lower CFUs than infections with CC3 or CC13^50^. Nevertheless, the results of our study, which included antibiotic interventions 2 days after grafting—the I0A2 groups—demonstrated the efficacy of OTC, TYL, and LMC in treating infected larva in vitro.

## Conclusion

In summary, we identified, for the first time, an OTC-resistant isolate responsible for EFB disease in a North American apiary. *M. plutonius* 2019BC1 remains susceptible to TYL and LMC, suggesting that these antimicrobials may be suitable treatments for colonies showing clinical signs of EFB disease due to infection with *M. plutonius* 2019BC1, however, further evaluation is required for approval in Canadian apiculture. In fact, we provide evidence to support further evaluation towards regulatory approval of TYL and LMC, along with OTC, as treatments for OTC-resistant *M plutonius* infection in honey bee larvae. The overlapping molecular target of all three antibiotics, however, remains a significant concern, especially considering the lack of alternative treatment options. Additional research is required to characterize the AMR phenotypes and genotypes of *M. plutonius* strains circulating in North America where antibiotic use is common.
